# There is no clear link between muscle strength and depression, as the latter is multifactorial

**DOI:** 10.1590/1806-9282.20261191

**Published:** 2026-11-09

**Authors:** Carla Alexandra Scorza, Fulvio Alexandre Scorza, Josef Finsterer

**Affiliations:** 1 Universidade Federal de São Paulo – São Paulo (SP), Brazil.; 2 Neurology & Neurophysiology Center, Department of Neurology – Vienna, Austria.

Dear Editor,

We read with interest the review article by Sousa et al. regarding the association between muscle strength and depression or anxiety in older adults^1^. An inverse relationship was observed between muscle strength - specifically handgrip strength - and depression or anxiety^1^. While the study provides valuable insights, it warrants further discussion.

First, it should be noted that the mental health of older adults depends not only on physical fitness but also on numerous other factors. These include the presence or absence of chronic diseases (e.g., heart disease, cancer, Parkinson's disease, diabetes); cognitive function (cognitive decline causes anxiety and distress); number of social contacts (a lack of regular contact leads to loneliness and depression); the death of friends, family members, or spouses; the burden of caring for relatives; financial instability; lifestyle; life events; personality type; genetic predisposition; job loss; and environmental factors^2^. Unless these influencing factors are included in the analysis, the relationship between muscle strength and depression remains unclear.

Secondly, grip strength cannot be used as a benchmark for assessing general physical fitness. A patient who relies on a wheelchair may possess normal grip strength yet be unable to stand or walk. Furthermore, there are patients with polyneuropathy who exhibit normal muscle strength in the upper extremities but suffer from weakness or sensory deficits in the lower extremities. More comprehensive methods are required to assess general muscle strength. General muscle strength can be evaluated clinically using the muscular research council grading system, gravity tests, complex movement sequences (such as push-ups, squats, or rising from a chair without using one's hands), and indirect indicators (e.g., walking on heels or tiptoes, Gowers’ sign), as well as by means of hand dynamometers or isokinetic devices^3^.

Thirdly, older adults often present with multimorbidity and are consequently susceptible to muscle weakness - whether as a result of a systemic disease or as a side effect of chronic medication. Muscle strength can therefore depend heavily on the presence of comorbidities and current medication use. It should also be noted that normal muscle strength does not rule out the presence of fatigue, tiredness, exhaustion, or limited exercise tolerance^4^. A patient with heart failure may possess normal muscle strength yet still experience difficulty climbing stairs. Since a specific level of muscle strength does not necessarily indicate whether a person is regularly physically active, it is more meaningful to relate the intensity and type of physical activity to depression and anxiety than to a specific level of muscle strength.

A fourth point is that the assumption that muscle-strengthening interventions could improve mental health is not supported by the reported results^1^. The underlying study aimed to investigate whether a certain level of muscle strength correlates with the severity of depression or anxiety; however, it did not examine whether regular physical activity has antidepressant or anxiolytic effects. Studies with an appropriate design are needed to determine whether a specific type of muscle training has antidepressant or anxiolytic effects.

## Data Availability

The datasets generated and/or analyzed during the current study are available from the corresponding author upon reasonable request.
